# Association of Muscle Fibers with Histopathology in Doughnut Specimens Following Stapled Hemorrhoidopexy and Their Impacts on Postoperative Outcomes

**DOI:** 10.1055/s-0042-1743520

**Published:** 2022-08-24

**Authors:** Chetty Y. V. Narayanaswamy, M. R. Sreevathsa, G. Akhil Chowdari, Koteshwara Rao

**Affiliations:** 1 Department of General Surgery, Ramaiah Medical College, Mathikere, Bangaluru, Karnataka, India

**Keywords:** anus, hemorrhoids, stapled hemorrhoidopexy

## Abstract

**Background** Stapled hemorrhoidopexy is widely practiced worldwide since its introduction to the world of proctology and replaced conventional hemorrhoidectomy in treating hemorrhoids. The technique of executing the procedure dictates the outcomes and complications. Here, we attempted to establish the cause of postoperative complications and attributed them to the presence of muscle of fibers in the excised doughnut specimens.

**Materials and Methods** A prospective observational analysis of histopathological specimens obtained from patients who underwent stapled hemorrhoidopexy using procedure for prolapse and hemorrhoids-03 circular staplers in the department of surgery of a tertiary care hospital in southern India was performed, and the correlation between the presence or absence of muscle fibers in the specimens and postoperative complications was evaluated. The patients were followed up for 12 months after the procedure.

**Results** In this study, 155 patients, including 54, 91, and 10 patients with Grade 2, Grade 3, and Grade 4 hemorrhoids, respectively, were included. Group A consisted of 19 patients with muscle fibers on the specimens, whereas Group B consisted of 139 patients without muscle fibers on the specimens. Early complications within 7 days after the procedure were as follows: 21 and 0.7% of the patients in Groups A and B, respectively, presented with postoperative pain with a visual analog scale score of more than 4; 47 and 6% of the patients in Groups A and B, respectively, presented with urinary retention; 26 and 2% of the patients in Groups A and B, respectively, presented with bleeding; and 21 and 2.9% of the patients in Groups A and B, respectively, presented with fecal urgency. A significant association was found between the presence of muscle fibers and early complications ( *p* < 0.001). Late complications, such as proctalgia and bleeding, accounting for 36.8 and 6.6% in Groups A and B, respectively, were significantly associated with the presence of muscle fibers in histopathology ( *p* < 0.001). Meanwhile, other late complications, such as incontinence, stenosis, and recurrence, exhibited no association ( *p* > 0.05).

**Conclusion** The technique in taking purse-string sutures and the depth of the suture bite above the dentate line carry the utmost importance in preventing postoperative complications. Therefore, surgeons should refine their technique of appropriate depth to avoid incorporation of muscle fibers while executing the procedure.

Hemorrhoids are a common surgical condition, accounting for ∼75% of the Indian population, 1 and they present with symptoms of bleeding, prolapse, itching, pain, and mucous discharge. For early grades of hemorrhoids, conservative treatment alone or in association with rubber band ligation showed good treatment outcomes. Additionally, other treatment options for hemorrhoids, such as infrared coagulation, sclerotherapy, laser therapy, and cryosurgery, have been used occasionally. However, for higher grade hemorrhoids, surgery remains the first choice of treatment.

In 1888, the founder of St. Mark's Hospital, Fredrich Solomon, proposed a combination of excision and ligation of hemorrhoids. Following this, this technique has seen several modifications with traditional surgery, such as the Fergusson method, the Milligan–Morgan technique, and diathermy hemorrhoidectomy in an attempt to reduce postoperative pain with limited degree of success. In 1998, Longo described procedure for prolapse and hemorrhoids (PPH), a technique for stapled hemorrhoidectomy, which is performed using a specially designed stapler. 2 The rationale for this procedure is stapled resection of complete circular strip of the mucosa above the dentate line, lifting hemorrhoidal cushions (anopexy), which restores the anatomical and physiological anatomy of the hemorrhoidal plexus. 3 PPH was received with enthusiasm, as it could be executed with speed and result in less postoperative pain and good postoperative outcomes. This technique has become an alternative to hemorrhoidectomy, which can be reproduced without any subjective variability. 4 5

However, many studies on postoperative outcomes and complications of stapled hemorrhoidectomy have been published. 6 Despite constant modifications and refinement of the stapler gun, this technique has fallen in repute because of the errors by the man behind the machine or occasionally the machine itself, resulting in both early and late complications. Additionally, some rare and life-threatening complications of this procedure have been reported.

Stapled hemorrhoidopexy has been described to exclude the sphincter muscle during purse-string application. The literature suggests that smooth muscle fibers should be absent in ideal specimens. 7 Much emphasis on the technique of the procedure has been explained by many authors, especially in taking the purse-string stitch relative to the dentate line and the depth of the bites, which determines the outcome of this procedure.

Hence, this study was conducted to determine the association between postoperative complications and the presence of muscle fibers on specimens as an indirect indicator of doughnut depth.

## Materials and Methods

This was a prospective observational study conducted between October 2016 and March 2018 at territory care hospital, Bengaluru. After obtaining the ethical clearance certificate from the Institutional Ethical Review Board, convenient sampling method was used for sample size estimation. Patients with symptoms of hemorrhoids were evaluated in the outpatient department. History taking, physical examination, and proctoscopy were performed in all patients. The degree of hemorrhoidal disease was evaluated using the following grading system: Grade 1, dilated blood vessels; Grade 2, prolapsed but spontaneously reducible piles; Grade 3, piles that required manual reduction; and Grade 4, permanently prolapsed hemorrhoids. 8 Colonoscopy was recommended and performed in selected cases before surgery. Patients older than 18 years with Grade 2 hemorrhoids not responding to conservative and day care procedures and Grades 3 and 4 hemorrhoids were included in the study. However, Grade 4 hemorrhoids patients were offered other modalities of treatment for their condition and explained the outcome of each procedure, and it was their choice to undergo stapler hemorrhoidopexy. Patients with acute prolapsed hemorrhoids, thrombosed piles, bulky hemorrhoids, fistula-in-ano, fissure-in-ano, recurrent disease, lax sphincter tone, prior anorectal surgery, local radiation, and malignancy were excluded from the study. The aforementioned conditions could cause fibrosis in the submucosal layer resulting in inadvertent sequestration of deeper tissue into the doughnut, which could alter postoperative outcomes. Approximately 236 patients who met the aforementioned criteria were offered a choice between conventional hemorrhoidectomy and stapled hemorrhoidopexy following education on the degree and duration of postoperative pain with each method and the expenditure involved. Of the 236 patients, 155 chose to undergo stapled hemorrhoidopexy, and the remaining patients chose to undergo conventional hemorrhoidectomy. Surgery was performed as an inpatient procedure with enema administered 10 hours before surgery. A single dose of antibiotic was administered at the beginning of surgery in all patients. Hemorrhoidopexy was performed in the lithotomy position under spinal anesthesia using a PPH-03 circular stapler (Ethicon, Inc., NJ). The purse-string suture technique was performed using half-circle round body 2–0 Prolene on the area 2 cm above the hemorrhoidal pedicle and at least 4 cm cranial to the dentate line. The doughnut specimen was examined for completeness and submitted for histopathological examination with due special consent. The pathologist was requested to report the depth of the doughnut, especially for the presence or absence of muscle fibers in the given specimen. The patients were discharged if the following criteria were met: postoperative defecation without a relevant amount of blood in the stool and good pain control with minimum use of pain killers. Stool softeners were recommended in all patients after surgery. The patients were classified into two groups based on the presence or absence of muscle fibers on histopathological examination of doughnut specimens. Group A consisted of patients with muscle fiber, whereas Group B comprised patients without muscle of fiber in the doughnut specimens, and these groups were observed for early and late complications.

Early complications were defined as complications occurring within 7 days after the operation, whereas late complications were defined as those occurring after 7 days. Patients were followed up on postoperative days 7 and 14 and at the end of months 1, 6, and 12.

Statistical software R 4.0.3 was used for analysis. To see the association between two categorical variables, chi-square test was performed. Continues variables were represented as mean and standard deviation. Categorical variables were represented as frequency and percentage.

## Results

In this study, 155 patients who met the inclusion criteria were included. The mean age of the patients was 43.6 years. Among them, 57 were females, and 98 were males. Moreover, 54 patients (34.83%) presented with Grade 2 hemorrhoids, 91 patients (58.70%) presented with Grade 3 hemorrhoids, and the remaining 10 patients (6.45%) presented with Grade 4 hemorrhoids. Of the 155 patients, 40 presented with diabetes, 29 presented with hypertension, and 7 presented with ischemic heart disease as comorbidities.

The main early postoperative complication ( Table 1 ) was immediate postoperative pain where 83.22% exhibited tolerable pain with visual analog scale (VAS) scores of less than 4, 7% patients exhibited VAS scores of 4 to 6, and 9.67% exhibited VAS scores of ≥7. A significant reduction in pain scores was observed at the end of day 3 with 6.45% of the patients exhibiting VAS scores of 4 to 6 and at the end of day 7 with 3.22% of the patients exhibiting VAS scores of 4 to 6, though proctoscopic examination was unremarkable. Acute urinary retention was noted in 17 of the 155 patients (10.96%), which required temporary indwelling catheterization; no patient exhibited urosepsis or chronic urinary retention with significant morbidity. Other early complications included postoperative bleeding, which was observed in eight patients (5.16%) of whom three required packing using absorbable hemostatic gelatin sponges. No patient required blood transfusion or resurgery as all patients recovered spontaneously. Eight patients (5.16%) exhibited fecal urgency, which resolved at the end of day 7 as postoperative pain gradually decreased.

**Table 1:** Early postoperative complications

	Male ( *n* = 98)			Female ( *n* = 57)			Total ( *n* = 155)			Percentage
	Day 1	Day 3	D	Day 1	Day 3	Day 7	Day 1	Day 3	Day 7	
Pain VAS <4	80	91	94	49	54	56	129	145	150	
Pain VAS 4–6	8	7	4	3	3	1	11	10	5	
Pain VAS ≥ 7	10			5			15			
Urinary retention										
Present	13			4			17			10.96%
Absent	85			53			138			
Bleeding										
Present	5			3			8			5.16%
Absent	93			54			147			
Fecal urgency										
Present	6			2			8			5.16%
Absent	92			55			147			

Abbreviation: VAS, visual analog scale.

Sixteen patients (10.32%) presented with proctalgia, characterized by continuous pain localized at the anus with aggravation during defecation, which is responsive to oral analgesics. On subjecting these patients to proctoscopy, no clinical evidence of submucous hematoma, abscess, and granuloma was noticed. This intensity of pain at times had compromised normal lifestyle and reintegration of work. These patients required long-term analgesic medications with stool softeners and high-fiber diet, and the symptoms subsided 3 to 4 months after surgery further avoiding for any clinical–radiological investigations. Moreover, 16 patients (10.32%) exhibited intermittent late postoperative bleeding, and in 5 patients (3.22%), hemorrhoids recurred 4 to 6 months after surgery, 2 of whom underwent rubber band ligation, 1 underwent open conventional hemorrhoidectomy, and 2 refused surgical intervention and opted for conservative management. Note that four of the five patients with recurrence exhibited Grade 4 hemorrhoids. Other complications were noted in negligible numbers, including mild incontinence in two patients (1.29%) and stenosis in two patients (1.29%). Patients with incontinence improved with pelvic floor exercises, and those with stenosis improved with simple gradual dilatation with serial dilators ( Table 2 ).

**Table 2:** Late complications

	Male ( *n* = 98)	Female ( *n* = 57)	Total ( *n* = 155)	Percentage
Proctalgia				
Present	9	7	16	10.32%
Absent	89	50	139	
Bleeding				
Present	10	6	16	10.32%
Absent	88	51	139	
Recurrence				
Present	3	2	5	03.22%
Absent	95	55	150	
Incontinence				
Present	1	1	2	01.29%
Absent	98	55	153	
Stenosis				
Present	1	1	2	01.29%
Absent	97	56	153	

### Correlation of Postoperative Complications with the Presence of Muscle Fibers on Histopathology of Doughnut Specimens Following Stapled Hemorrhoidopexy

**Fig. 1:**
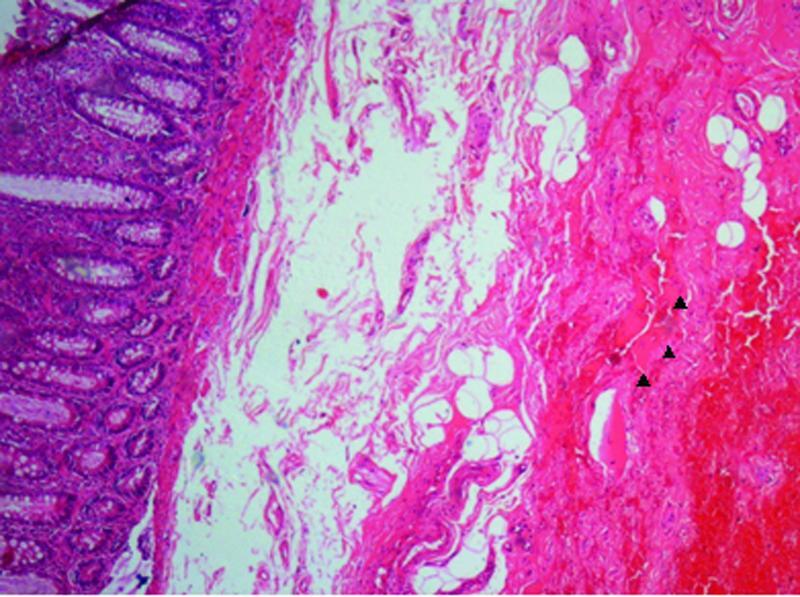
High power magnification of histopathology of a doughnut specimen showing muscle fibers (arrow head).

**Fig. 2:**
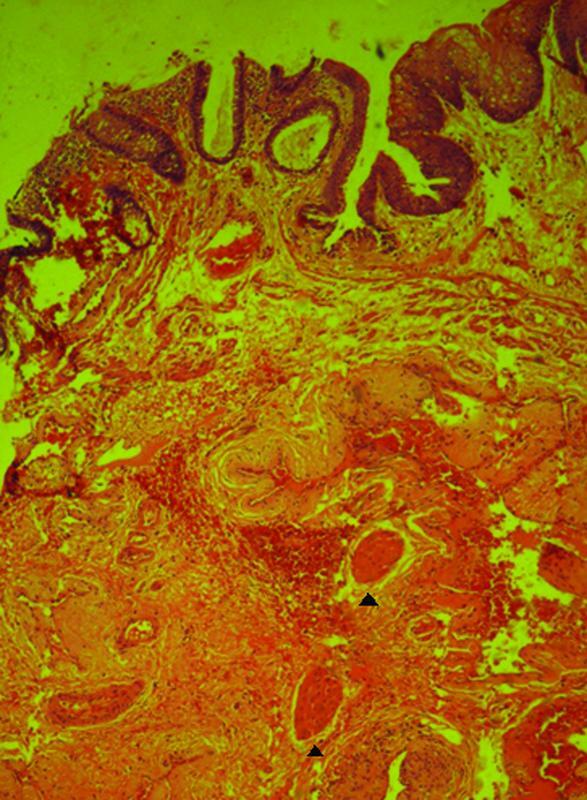
Low power magnification of histopathology of a doughnut specimen showing muscle fibers (arrow head).

On histopathological examination of the doughnut specimens obtained from the 155 patients, 19 patients (12.25%) exhibited muscle fibers indicating that deeper tissue was involved in the resected specimen ( Figs. 1 and 2 ). The postoperative complications in Group A were compared with those in Group B.

Regarding early complications with the presence of muscle fibers in histopathology examination ( Table 3 ), 16 patients in Group A exhibited pain scores of more than 4 on day 1, whereas 10 patients in Group B exhibited pain scores of more than 4. On day 3, 7 of the 19 patients in Group A and 3 of the 136 patients in Group B exhibited pain scores of more than 4, whereas on day 7, 4 of the 19 patients in Group A and 1 of the 136 patients in Group B exhibited pain scores of more than 4. The aforementioned differences were statistically significant ( *p* ≤ 0.001). Additionally, urinary retention was observed in nine patients in Group A and eight patients in Group B, and fecal urgency was noted in four patients in Group A and four patients in Group B, whereas early postoperative bleeding was found in five patients in Group A and three patients in Group B. The differences in early complications between the two groups were statistically significant ( *p* ≤ 0.001).

**Table 3:** Correlation of early complications with the presence of muscle fibers in HPE

	Presence of muscle fibers in HPE			Absence of muscle fibers in HPE			*p* -Value
	Group A ( *n* = 19)			Group B ( *n* = 136)			
	VAS <4	VAS 4–6	VAS ≥7	VAS <4	VAS 4–6	VAS ≥7	
Pain day 1	3	7	9	126	4	6	<0.001
Pain day 3	12	7	0	133	3	0	<0.001
Pain day 7	15	4	0	135	1	0	0.001
Urinary retention	9 (47%)			8 (5.88%)			<0.001
Bleeding	5 (26.31%)			3 (2.20%)			<0.001
Fecal urgency	4 (21.05%)			4 (2.94%)			0.001

Abbreviations: HPE, histopathology examination; VAS, visual analog scale.

Regarding late complications ( Table 4 ), seven patients in Group A and nine patients in Group B exhibited proctalgia, and a similar number of cases exhibited bleeding. The differences in late complications between the two groups were statistically significant ( *p* < 0.001). No association was found between the two groups in terms of recurrence, incontinence, and stenosis ( *p* > 0.05).

**Table 4:** Correlation of late complications with the presence of muscle fibers in HPE

	Presence of muscle fibers in HPE	Absence of muscle fibers in HPE	*p* -Value
	Group A ( *n* = 19)	Group B ( *n* = 136)	
Proctalgia	7 (36.84%)	9 (6.61%)	<0.001
Bleeding	7 (36.84%)	9 (6.61%)	<0.001
Recurrence	2 (10.52%)	3 (2.2%)	0.055
Incontinence	1 (5.26%)	1 (0.73%)	0.101
Stenosis	1 (5.26%)	1 (0.73%)	0.101

Abbreviation: HPE, histopathology examination.

### Correlation of Complications with Respect to Grade of Hemorrhoids and Muscle Fiber Presence on Histopathology

Grade 2 hemorrhoids ( Table 5 ) showed association between the groups only for pain on postoperative day 1 ( *p* < 0.05) and rest all complications were statistically insignificant.

**Table 5:** Association of muscle fiber on early and late complications in Grade 2 group

Early complications		Muscle fiber in HPE		*p* -Value
		Present [ *N* = 3] (%)	Absent [ *N* = 51] (%)	
Pain day 1 (VAS)	< 4	1 (33.33)	47 (92.16)	0.032*
	4–7	1 (33.33)	2 (3.92)	
	≥ 7	1 (33.33)	2 (3.92)	
Pain day 3 (VAS)	< 4	3 (100)	51 (100)	–
Pain day 7 (VAS)	< 4	3 (100)	51 (100)	–
Urinary retention	Present	1 (33.33)	2 (3.92)	0.152
	Absent	2 (66.67)	49 (96.08)	
Bleeding	Present	0	1 (1.96)	0.999
	Absent	3 (100)	50 (98.04)	
Fecal urgency	Absent	3 (100)	51 (100)	–
**Late complications**		**Present**	**Absent**	***p*** **-Value**
Proctalgia	Present	0	3 (5.88)	0.999
	Absent	3 (100)	48 (94.12)	
Bleeding	Present	0	3 (5.88)	0.999
	Absent	3 (100)	48 (94.12)	
Incontinence	Absent	3 (100)	51 (100)	–
Recurrence	Absent	3 (100)	51 (100)	–
Stenosis	Absent	3 (100)	77 (100)	–

Abbreviations: HPE, histopathology examination; VAS, visual analog scale.

While evaluating for Grade 3 hemorrhoids ( Table 6 ), there was statistical association between pain on postoperative days 1 and 2, urinary retention, and bleeding in early complications ( *p* < 0.05) and proctalgia and bleeding in late complications ( *p* < 0.05), whereas Grade 4 hemorrhoids ( Table 7 ) had only one parameter, postoperative pain on day 7, in early complications with significant association.

**Table 6:** Association of muscle fiber on early and late complications in Grade 3 group

Early complications		Muscle fiber in HPE		*p* -Value
		Present [ *N* = 14] (%)	Absent [ *N* = 77] (%)	
Pain day 1 (VAS)	< 4	2 (14.29)	74 (96.10)	<0.0001 ^a^
	4–7	6 (42.86)	1 (1.30)	
	≥ 7	6 (42.86)	2 (2.60)	
Pain day 3 (VAS)	< 4	9 (64.29)	75 (97.40)	0.001 ^a^
	4–7	5 (35.71)	2 (2.60)	
Pain day 7 (VAS)	< 4	12 (85.71)	76 (98.70)	0.06
	4–7	2 (14.29)	1 (1.30)	
Urinary retention	Present	6 (42.86)	4 (5.19)	0.001 ^a^
	Absent	8 (57.14)	73 (94.81)	
Bleeding	Present	3 (21.43)	1 (1.30)	0.014 ^a^
	Absent	11 (78.57)	76 (98.70)	
Fecal urgency	Present	2 (14.29)	2 (2.60)	0.126
	Absent	12 (85.71)	75 (97.40)	
**Late complications**		**Present**	**Absent**	***p*** **-Value**
Proctalgia	Present	5 (35.71)	3 (3.90)	0.001 ^a^
	Absent	9 (64.29)	74 (96.10)	
Bleeding	Present	5 (35.71)	3 (3.90)	0.001 ^a^
	Absent	9 (64.29)	74 (96.10)	
Incontinence	Present	0	1 (1.30)	0.999
	Absent	14 (100)	76 (98.70)	
Recurrence	Present	0	1 (1.30)	0.999
	Absent	14 (100)	76 (98.70)	
Stenosis	Absent	14 (100)	77 (100)	–

Abbreviations: HPE, histopathology examination; VAS, visual analog scale.

**Table 7:** Association of muscle fiber on early and late complications in Grade 4 group

Early complications		Muscle fiber in HPE		*p* -Value
		Present [ *N* = 2] (%)	Absent [ *N* = 8] (%)	
Pain day 1 (VAS)	< 4	0	5 (62.50)	0.354
	4–7	0	1 (12.50)	
	≥ 7	2 (100)	2 (25.00)	
Pain day 3 (VAS)	< 4	0	7 (87.50)	0.068
	4–7	2 (100)	1 (12.50)	
Pain day 7 (VAS)	< 4	0	8 (100)	0.023 ^a^
	4–7	2 (100)	0	
Urinary retention	Present	2 (100)	2 (25.00)	0.139
	Absent	0	6 (75.00)	
Bleeding	Present	2 (100)	1 (12.50)	0.065
	Absent	0	7 (87.50)	
Fecal urgency	Present	2 (100)	2 (25.00)	0.139
	Absent	0	6 (75.00)	
**Late complications**		**Present**	**Absent**	***p*** **-Value**
Proctalgia	Present	2 (100)	3 (37.50)	0.440
	Absent	0	5 (62.50)	
Bleeding	Present	2 (100)	3 (37.50)	0.440
	Absent	0	5 (62.50)	
Incontinence	Present	1 (50.00)	0	0.198
	Absent	1 (50.00)	8 (100)	
Recurrence	Present	2 (100)	2 (25.00)	0.139
	Absent	0	6 (75.00)	
Stenosis	Present	1 (50.00)	1 (12.50)	0.369
	Absent	1 (50.00)	7 (87.50)	

Abbreviations: HPE, histopathology examination; VAS, visual analog scale.

## Discussion

Hemorrhoids are usually considered one of the most common anorectal disorders. Patients often tend to hesitate to seek treatment resulting in disease progression due to social taboos associated with hemorrhoids.

PPH first described by Longo in 1998 was perceived globally due to its advantages, and many case series, meta-analyses, and systemic reviews showed that it is both safe and effective and is better than conventional hemorrhoidectomy in terms of postoperative pain and early return to work. 9 10 However, in recent studies, long-term follow-up assessing complications of stapled hemorrhoidectomy led to suspicion surrounding the safety and cost-effectiveness of this procedure compared with other techniques available for hemorrhoidectomy. 11 In a systemic review conducted by Porrett et al 6 where 78 studies with follow-up periods ranging from 1 month to 7 years, involving 14,234 patients, researchers reported postoperative complications of stapled hemorrhoidectomy, the incidence rates of early complications widely ranged between 2.3 and 52.5% with a median value of 16.1%, and the incidence rates of late complications ranged between 2.5 and 80% with a median value of 23.7%. The aforementioned studies also reported that the cause of complications could be attributed to the presence of muscle fibers in the resected doughnut.

In a study conducted by Stolfi et al, 4 where stapled hemorrhoidectomy was compared with the Milligan–Morgan method in 200 patients (100 patients in each group), no difference in postoperative pain was observed between the two groups on the first 2 days, but in the next 6 consecutive days, patients who received stapled hemorrhoidectomy demonstrated significantly less pain. 12 In this study, a similar pattern of pain scores was observed with only five (3.2%) patients presenting with residual pain on postoperative day 7 requiring analgesics for pain control. However, an association was found between higher pain scores and the presence of muscle fibers on histopathology, and this association was statistically significant.

Urinary retention is a common complication after anorectal surgery with rates ranging from 3 to 50%. Most studies reported a rate of 15%. Postoperative urinary retention is multifactorial with contributions from irritation/blockade of pelvic nerves and pain-evoked reflexes. Ommer et al 13 reported similar observations. In a study conducted by Sultan et al involving more than 150 patients, despite exhibiting a postoperative morbidity rate of as low as 9.3% and high patient satisfaction in the postoperative period following stapled hemorrhoidopexy, acute urinary retention accounted for 7.3%. 5 In this study, 10.96% of the patients exhibited urinary retention; however, the incidence of urinary retention in the patients presenting with muscle fibers in histopathology was ∼47%.

The incidence of early postoperative bleeding ranges from 4.2 to 7.5%. 14 This complication is commonly due to arteriolar bleeding along the staple line or folding of excessive mucosa into the staple line. This study reported an incidence rate of 5.16%, and early bleeding was significantly associated with the presence of muscle fibers in histopathology examination. In 2010, Sultan et al observed that using staples with a smaller staple bite of 0.75 to 1.5 mm (PPH-03), instead of its precedent of 1.2 to 2.5 mm (PPH-01), led to better compression of rectal tissue and bleeding vessels, reducing the incidence of early postoperative bleeding. 15

The reported incidence rates of early fecal urgency range from 0 to 25% with a median of 8.28%. 16 The etiology of this complication could be attributed to muscle contraction in response to nerve or muscle irritation, which may decrease rectal compliance. Fecal urgency usually disappears in the first few weeks after surgery in most cases. 5 In this study, 5.16% of the patients presented with fecal urgency with spontaneous recovery by the end of the first postoperative week, and this complication was associated with the presence of muscle fibers on histopathologically examination.

Most complications are either related to improper selection of cases for surgery or technical errors either by the man behind the machine or the machine itself. The earliest study was conducted in year 2000 by Cheetham et al involving 22 cases of stapled hemorrhoidectomy, including 7 cases of pilot study and 15 cases of randomized controlled study with open hemorrhoidectomy. They observed persistent severe pain and fecal urgency in several patients after stapled hemorrhoidectomy, followed by conventional open surgery. The mechanism behind the complications was unclear, and muscle fiber incorporation in the doughnut may have contributed. 17 Similar studies by Ielpo et al reported that 14.3% of patients exhibited pain lasting more than 2 weeks. They correlated that this postoperative pain to additional hemostatic stitches, causing ischemic pain, and endoanal ultrasound demonstrated retained staples with inflammation around them. Thus, Ielpo et al recommended carefully applying hemostatic stitches to avoid inclusion of muscle fibers and complete burrowing the staples. 18

Postoperative pain is determined by both the ideal placement of suture in relation to the dentate line and the depth of purse-string suture taken. In a study by Plocek et al in 2006, the height of staples seemed to be correlated with the duration of narcotic pain management and interval to return to work. 19 In 2007, Ganio et al observed that the staple line being well above the dentate line is the cause of elevated sphincter muscle tone, causing chronic pain in the postoperative period. 20 The incorporation of smooth muscles into the doughnut and the induction of the inflammatory process in the staple line may increase the incidence of postoperative pain. The formation of fibrosis near the staple line may chronically stimulate the nerve spindles located over the puborectalis muscle, causing pudendal neuropathy with chronic proctalgia and fecal urgency. 4

The cause for late bleeding, which occurs usually between postoperative weeks 6 and 16, may be attributed to inflammation around the staple line or its rejection or the presence of inflammatory polyps along the staples, requiring local remedial measures. 15 In this study, we found that late postoperative bleeding and proctalgia were found in 10.32% of the patients, and the difference in the incidence of these complications between patients with and without muscle fibers was statistically significant.

The recurrence rate following stapled hemorrhoidopexy was 11%, with ∼10% of patients requiring reoperation. 21 The recurrence rate after stapled hemorrhoidopexy (8.5%) was significantly higher than that after manual hemorrhoidectomy (1.5%). 22 Riss et al conducted a long-term study with a mean follow-up duration of 48 months and reported that Longo's technique of stapled hemorrhoidopexy demonstrated no negative impact with significant improvements in evacuation scores. 23 In 2013, Hong et al concluded that the incorporation of muscle fibers in the resected doughnuts after stapled hemorrhoidopexy may affect anorectal manometry results; however, they did not find any significant differences in the postoperative outcomes. 24 The incidence of stenosis after stapled hemorrhoidopexy ranges from 0 to 8%, and most cases resolve with conservative management (i.e., outpatient dilatation), rarely requiring surgical intervention. 25 In this study, we found that the rate of recurrence was 3%, the rate of incontinence was 2%, and the rate of stenosis was less than 1.5%. When comparing Group A with Group B, the results were statistically insignificant.

In November 2002, George et al conducted a study examining the histopathology of resected specimens obtained from 26 consecutive patients who underwent stapled hemorrhoidectomy. 26 The specimens were examined for the type of mucosa and muscle fibers in detail and observed for clinical outcomes postoperatively. They concluded that the level of purse-string suture and depth determined the presence of stratified epithelium or internal anal muscle fibers, and surgeons should be aware of the importance of the technique to avoid injury to the internal anal sphincter.

Some life-threatening early complications have been documented following stapled hemorrhoidopexy, such as pelvic sepsis, rectal perforation, Fournier's gangrene, acute hemorrhage, rectopneumoperitoneum, rectal stricture, and rectovaginal fistula, which brought disrepute to this technique compared with other available modalities. 27 Most complications were attributed to technical error or wrong indications.

While including the grade of hemorrhoids for statistical evaluation, the inclusion of muscle fiber was pivotal in giving rise to complications for Grade 3 hemorrhoids patients and not much of impact for Grades 2 and 4 hemorrhoids.

With careful selection of cases and proper technique execution, stapled hemorrhoidopexy remains to be an accepted modality for treating hemorrhoids. 28 The overall complication rates in this study were low, accounting for 16.7% of the patients among whom 61.5% exhibited muscle fibers in the doughnut specimens. Additionally, early complications were significantly associated with the presence of muscle fibers. No long-term residual or life-threatening postoperative complications were observed in this study.

Endorectal ultrasound evaluation would have given accurate analysis of fragmentation of anorectal muscles, however, due to the lack of facilities for endorectal ultrasound in the institution is the drawback of this study.

## Conclusion

The inclusion of muscle fibers while executing stapled hemorrhoidopexy can lead to complications especially in Grade 3 hemorrhoids. The technique of taking the purse-string sutures is purely subjective and surgeon dependent. Sending histopathology specimens would provide feedback to the operating surgeon for further refining the technique of taking purse-string sutures with optimal depth to avoid inadvertent inclusions of muscle fibers.
